# Silencing of NAC1 Expression Induces Cancer Cells Oxidative Stress in Hypoxia and Potentiates the Therapeutic Activity of Elesclomol

**DOI:** 10.3389/fphar.2017.00804

**Published:** 2017-11-07

**Authors:** Yi-Jie Ren, Xiao-Hui Wang, Cheng Ji, Yi-Di Guan, Xian-Jiu Lu, Xian-Rong Liu, Hong-Han Zhang, Ling-Chuan Guo, Qiong-Hua Xu, Wei-Dong Zhu, Zhi-Jun Ming, Jin-Ming Yang, Yan Cheng, Yi Zhang

**Affiliations:** ^1^Department of Pharmacology, College of Pharmaceutical Sciences and Department of Respiratory Medicine, First Affiliated Hospital, Soochow University, Suzhou, China; ^2^Department of Pharmacology, School of Pharmaceutical Sciences, Central South University, Changsha, China; ^3^Department of Gastrointestinal Surgery, Affiliated Nanhua Hospital, University of South China, Hengyang, China; ^4^Penn State Hershey Cancer Institute, The Pennsylvania State University College of Medicine, Hershey, PA, United States

**Keywords:** NAC1, oxidative stress, PDK3, hypoxia, elesclomol

## Abstract

In order to survive under conditions of low oxygen, cancer cells can undergo a metabolic switch to glycolysis and suppress mitochondrial respiration in order to reduce oxygen consumption and prevent excessive amounts of reactive oxygen species (ROS) production. Nucleus accumbens-1 (NAC1), a nuclear protein of the BTB/POZ gene family, has pivotal roles in cancer development. Here, we identified that NAC1-PDK3 axis as necessary for suppression of mitochondrial function, oxygen consumption, and more harmful ROS generation and protects cancer cells from apoptosis in hypoxia. We show that NAC1 mediates suppression of mitochondrial function in hypoxia through inducing expression of pyruvate dehydrogenase kinase 3 (PDK3) by HIF-1α at the transcriptional level, thereby inactivating pyruvate dehydrogenase and attenuating mitochondrial respiration. Re-expression of PDK3 in NAC1 absent cells rescued cells from hypoxia-induced metabolic stress and restored the activity of glycolysis in a xenograft mouse model, and demonstrated that silencing of NAC1 expression can enhance the antitumor efficacy of elesclomol, a pro-oxidative agent. Our findings reveal a novel mechanism by which NAC1 facilitates oxidative stress resistance during cancer progression, and chemo-resistance in cancer therapy.

## Introduction

In order for tumor cells to adapt to low oxygen conditions, elevated glucose uptake and increased conversion to lactate often occur. This phenomenon is called the Pasteur Effect. It is considered that Pasteur Effect is result from glycolytic flux activation with mitochondrial respiration impairment for the lack of oxygen (Papandreou et al., 2006; Fukuda et al., 2007). A serious of reports demonstrated that this metabolic switch may favor the tumor cells survival in hypoxia through increasing lipids, nucleotides, and amino acids biosynthesis that are required for rapidly proliferating cells (Vander Heiden et al., 2009), leading to consistent acidification of extracellular surroundings and favoring the tumor cells acquisition of chemotherapy resistance and invasion (Annibaldi and Widmann, 2010), suppressing mitochondrial function to control oxygen consumption and preventing excessive amounts of reactive oxygen species (ROS) production thus inhibiting the occurrence of apoptosis (Kroemer and Pouyssegur, 2008).

Nucleus accumbens-1 (NAC1), is a cancer-related transcription factor belonging to the BTB/POZ (**b**ric-a-brac **t**ramtrack **b**road complex/**po**xvirus and **z**n finger) family (Nakayama et al., 2006; Perez-Torrado et al., 2006). The conserved BTB domain mediates NAC1 homo-dimerization, which is involved in various biological processes including maintenance of embryonic stem cells pluripotency and cancer pathogenesis (Nakayama et al., 2006; Wang et al., 2006). *NACC1*, which encodes NAC1, amplified region at ch19p13.2 in cancer was first observed in high-grade ovarian cancer. It was found that NAC1 overexpression is closely associated with early tumor recurrence (Nakayama et al., 2010; Yeasmin et al., 2012), and NAC1 has been appreciated as one of the top potential cancer driver genes (Shih et al., 2011). We and other groups have demonstrated that through its transcription-dependent or -independent manners, NAC1 can activate Gadd45 cell survival pathway (Nakayama et al., 2007; Jinawath et al., 2009), promote autophagic response to mediate resistance to cisplatin (Zhang et al., 2012b), disable cellular senescence to enhance tumor initiation and development (Zhang et al., 2012a), regulate cancer cell cytokinesis to facilitate their incessant cellular divisions (Yap et al., 2012), and induce fatty acid metabolism (Ueda et al., 2010).

Our previous observation showed that NAC1 can promote glycolysis in hypoxic tumor cells (Zhang et al., 2017). Nevertheless, the influence of NAC1 on mitochondrial oxidative respiration in hypoxia is not clear. Herein, we report the critical role of NAC1 in suppressing mitochondrial mass, oxygen consumption, excessive ROS production and inhibiting hypoxia-induced apoptosis. We show that suppression of mitochondrial respiration in hypoxia by NAC1 is mediated though inducing expression of pyruvate dehydrogenase kinase 3 (PDK3) by HIF-1α at the transcriptional level, thereby inactivating pyruvate dehydrogenase (PDH) and blocking the entrance of pyruvate into TCA cycle and the induction of mitochondrial oxidative respiration. Further, targeting of NAC1 for enhancing ROS production can improve the antitumor activity of elesclomol, a pro-oxidative agent.

## Materials and Methods

### Cell Lines and Cell Culture

The human ovarian cancer cell line SKOV3, the human cervical cancer cell line HeLa, were purchased from ATCC (Manassas, VA, United States). The identity of these cell lines was recently verified by STR analysis. HeLa cell line was cultured in DMEM medium supplemented with 10% heat-inactivated fetal bovine serum, 100 units/ml of penicillin and 100 mg/ml of streptomycin. SKOV3 cell line was cultured in RPMI 1640 medium supplemented with 10% heat-inactivated fetal bovine serum, 100 units/ml of penicillin and 100 mg/ml of streptomycin. Cells were cultured at 37°C in a humidified atmosphere of 20% O_2_/5% CO_2_ (normoxia) or 1% O_2_/5% CO_2_ (hypoxia). All cultures were monitored routinely and found to be free of contamination by mycoplasma or fungi. All cell lines were discarded after 3 months and new lines propagated from frozen stocks.

### siRNA, shRNA and Plasmid Transfection

Transfection of siRNA and plasmid was conducted using lipofectamine 2000 (Invitrogen, Carlsbad, CA, United States), according to the manufacturer’s protocol. NAC1 shRNA plasmid was synthesized by Santa Cruz, and transfection of shRNA plasmid was conducted following the manufacturer’s protocol. The transfected cells were selected with puromycin (5 μg/ml) for 2 weeks. Flag-PDK3 plasmid was purchased from QIAGEN. Transfection of the plasmid was carried out using lipofectamine 2000 (Invitrogen) according to the manufacturer’s protocol.

### RNA Isolation and Quantitative Real-Time PCR

Total RNA was prepared using TRIzol reagent (Roche, Basel, Switzerland). First-strand complementary DNA was synthesized using Omniscript reverse transcription kit (Qiagen, Hilden, Germany) with random primers. Quantitative reverse transcriptase-polymerase chain reaction (RT-PCR) was performed on ABI 7500 using Brilliant II SYBR Green QPCR master mix (Stratagene) and following primer sets: β*-actin*, 5′-GCCAACACAGTGCTGTCTGG-3′ (forward) and 5′-GCTCAGGAGGAGCAATGATCTTG-3′(reverse); *PDK1*, 5′-CTATGAAAATGCTAGGCGTCTGT-3′(forward) and 5′-AACCACTTGTATTGGCTGTCC-3′(reverse); *PDK2*, 5′-AGGACACCTACGGCGATGA-3′(forward) and 5′-TGCCGATGTGTTTGGGATGG-3′(reverse); *PDK3*, 5′-GCCAAAGCGCCAGACAAAC-3′ (forward) and 5′-CAACTGTCGCTCTCATTGAGT-3′(reverse); *PDK4*, 5′-TTATACATACTCCACTGCACCA-3′(forward) and 5′-ATAGACTCAGAAGACAAAGCCT-3′(reverse); After 40 cycles, data were collected and analyzed using the 7500 software (ABI, Waltham, MA, United States).

### Western Blotting and Antibodies

Cells were lysed in M-PER mammalian protein extraction reagent (Roche) supplemented with a cocktail of protease inhibitors (Roche, Indianapolis, IN, United States), followed by centrifugation at 14,000 × *g* for 10 min. After centrifugation, cell lysates were collected and protein concentrations were measured. Proteins (10–20 μg) were resolved by sodium dodecyl sulfate-polyacrylamide gel electrophoresis, and then transferred to PVDF membrane (Bio-Rad Laboratories, Hercules, CA, United States). Membranes were incubated with primary antibodies in 3% bovine serum albumin at 4°C for overnight, followed by incubation with secondary antibodies at room temperature for 1 h. The protein signals were detected by ECL (Beyotime Biotechnology, Shanghai, China) method. Antibodies to PDK3, PARP, caspase3, and β-actin were purchased from Santa Cruz Biotechnology Inc. (Santa Cruz, CA, United States); NAC1 antibody was obtained from Novus Biologicals LLC (Littleton, CO, United States).

### Infection Retrovirus PDK3 Construct

A retrovirus carrying the Flag-PDK3 expression vector was constructed by cloning the entire coding sequence of PDK3 into the pWZL-Hygro retroviral vector. NAC1 shRNA HeLa cells were infected with viruses for 48 h, followed by selection with Hygromycin (7.5 mg/mL for 2 weeks).

### Luciferase Reporter Assay

Two 18-base oligonucleotides (forward: 5′-CGCGTACGTGCAGCAACC-3′, reverse: 5′-CGCGTACGTGCAGAAACC-3′) corresponding to human PDK3 HRE matrix (-236/-219) were synthesized and cloned into SV40-driven pGL3 plasmid after annealing. Mutation of the putative HRE sequence was achieved by replacing the bases ACGTG with AAAAG, which was designed by RiboBio (Guangzhou, China). For the reporter assays, HeLa cells with silencing of NAC1 expression were transfected with or without Flag-HIF-1α were transfected with 1 μg of PDK3 reporter construct, pGL3-PDK3-Luc vector (wild type) or pGL3-PDK3-Luc vector (mutant), and 0.025 mg of PRL vector as an internal control, using FuGENE 6 transfection reagent. Twelve hours later, the cells were exposed to hypoxia for 24 h. Firefly and Renilla luciferase activities were measured using the Dual-Luciferase Reporter Assay system (Promega, Madison, WI, United States).

### NAO Staining

Cells were incubated in NAO-containing media (10 nM) for 30 min in the incubator, harvested, resuspended in PBS + 5% FCS (4°C) and analyzed by flow cytometry. A minimum of 10,000 events were analyzed using Guava EasyCyte Plus Flow Cytometry System (Millipore, Bedford, MA, United States). Treatment and staining of hypoxia-treated cells was performed with pre-equilibrated, hypoxic solutions.

### Oxygen Consumption Measurements

Cells were suspended at 2 × 10^6^ cells/ml in cell culture medium. Oxygen consumption was monitored with polarographic respirometry in an Oxygraph-2k (Oroboros) as described (Kluza et al., 2011) in full DMEM, glucose-free, or glutamine free medium +10% FCS at 37°C. Prior to collection of the cells by trypsinization, the cells were equilibrated for 1 h in the appropriate media type using hypoxic solutions for the hypoxic samples. The rate of oxygen consumption was normalized to cell numbers.

### Apoptosis Assay

Apoptosis was determined by flow cytometric analysis of Annexin V and 7-aminoactinomycin D staining. Briefly, 100 μl of Guava Nexin reagent (Millipore, Bedford, MA, United States) was added to 1 × 10^5^ cells in 100 μl, and the cells were incubated with the reagent for 20 min at room temperature in the dark. At the end of incubation, the cells were analyzed by a Guava EasyCyte Plus Flow Cytometry System (Millipore, Bedford, MA, United states).

### Mitochondrial Membrane Potential Assay

Mitochondrial membrane potential was determined with JC-1. Briefly, treated cells were collected and incubated with 1X JC-1 solution for 30 min at 37°C in a humidified atmosphere containing 5% CO_2_/95% air. At the end of incubation, fluorescence intensity of samples was analyzed on Infinite^®^ M1000 PRO (excitation at 485 nm and emission at 535 nm, TECAN, Switzerland).

### Detection of ROS

Reactive oxygen species generation was determined with DCFH-DA. Briefly, treated cells were collected and incubated with 10 μmol/L of DCFH-DA for 30 min at 37°C in a humidified atmosphere containing 5% CO_2_/95% air. At the end of incubation, fluorescence intensity of samples was analyzed on Infinite^®^ M1000 PRO (excitation at 488 nm and emission at 525 nm, TECAN, Switzerland).

### Cellular Viability Assay

Cell viability was measured by MTT assay. Briefly, cells were plated at 5 × 10^3^ cells per well in 96-well tissue culture plates and incubated at 37°C in a humidified atmosphere containing 5% CO_2_/95% air. The formazan product, formed after 4-h incubation with MTT, was dissolved in dimethyl sulfoxide and read at 570 nm on a Victor3 Multi Label plate reader (PerkinElmer).

### Measurement of PDH Activity

Pyruvate dehydrogenase activity was measured using PDH Assay Kit (State University of New York at Buffalo). Cells were lysed in the 10X sample buffer provided by the kit, 10 μl samples to a 96-well microplate and PDH activity was measured at 565 nm on a Victor3 Multi Label plate reader (PerkinElmer). The PDH activity was normalized to protein content.

### Imaging MicroPET Scan

Mice were anesthetized by Matrx VMR, and ^18^F-FDG (185 MBq/kg) was given through the tail vein to initiate emission scan. Images were acquired on microPET (Siemens Inveon, Siemens Healthcare, Erlangen, Germany). To quantify ^18^F-FDG uptake on the last frame (corresponding to 40–60 min), the obtained tissue activity [counts (kBq)/ml] was divided by the injected activity in kBq per gram of body weight (185 kBq/g) to give a standardized uptake value. Animal maintenance and experimental procedures were approved by the Institutional Animal Care and Use Committee of Soochow University.

### Animal Experiments

BALB/c athymic (nu/nu) mice (5-week-old, female) were inoculated subcutaneously with HeLa cells (4 × 10^6^ cells per mouse). One week after inoculation, the tumor-bearing mice were divided randomly into four groups (five mice per group): (1) control group; (2) NAC1 siRNA group; (3) Elesclomol group; and (4) NAC1 siRNA+Elesclomol group. Elesclomol (20 mg/kg) was given intravenously 5 days/week for 2.5 weeks. For delivery of cholesterol conjugated RNA, 10 nmol RNA in 0.1 ml saline was locally injected into the tumor mass q3d for 2.5 weeks. Tumor volumes were determined by measuring the length (L) and the width (W) of the tumors and calculating using the formula: V = L × W^2^/2. At the end of the experiment (on day 27), the mice were killed and tumors were surgically dissected. The tumor specimens were fixed in 4% paraformaldehyde for histopathologic examination. Animal maintenance and experimental procedures were approved by the Institutional Animal Care and Use Committee of Soochow University.

### Statistical Analysis

Statistical analyses were performed using Microsoft Excel software and GraphPad Prism. The results are presented as mean ± SD from at least three independent experiments. The *p*-values for comparisons between experimental groups were obtained by Student’s *t*-test. All statistical tests were two-sided. ^∗^*p* < 0.05, ^∗∗^*p* < 0.01.

## Results

### Silencing of NAC1 Expression Activates Mitochondrial Function in Hypoxia

**FIGURE 1:**
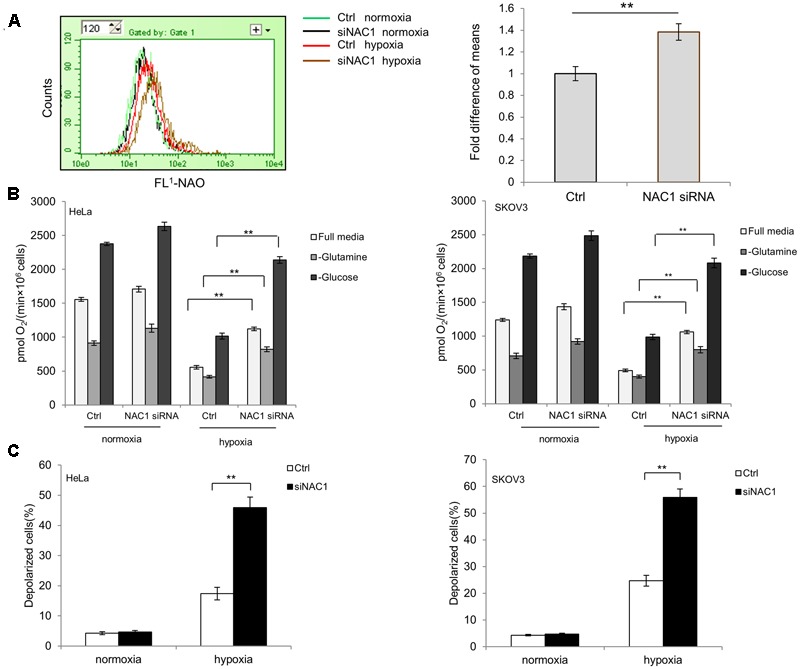
Silencing of Nucleus accumbens-1 (NAC1) expression activates mitochondrial function in hypoxic tumor cells. **(A)** HeLa cells transfected with a non-targeting RNA (Ctrl) or NAC1-targeted siRNA were incubated in normoxia or hypoxia (1% O_2_) for 24 h. Mitochondrial mass was estimated by nonyl acridine orange (NAO) staining. Fluorescence was measured with flow cytometry. Bars are mean ± SD representing the results for hypoxia-treated samples of three independent experiments. **(B)** HeLa and SKOV3 cells transfected with a non-targeting RNA (Ctrl) or NAC1-targeted siRNA were incubated in normoxia or hypoxia (1% O_2_) for 24 h. Oxygen consumption was measured in full, glucose-free, or glutamine-free media by using an oxygen electrode, and normalized to cell numbers. Bars are mean ± SD of three independent experiments. **(C)** HeLa and SKOV3 cells transfected with a non-targeting RNA (Ctrl) or NAC1-targeted siRNA were incubated in normoxia or hypoxia (1% O_2_) for 24 h. Mitochondrial membrane potential (ΔΨm) was assayed by JC-1 staining flow cytometry. Bars are mean ± SD of three independent experiments. ^∗∗^*p* < 0.01.

The impetus for this research arose from our previous observation that NAC1 can promote glycolysis of hypoxic tumor cells (Zhang et al., 2017). In order to determine whether the changes in glycolytic flux observed by loss of NAC1 would be accompanied by changes in mitochondrial function, we first evaluated the mitochondrial mass in the HeLa cells with or without silencing of NAC1 expression. **Figure 1A** shows that NAC1 silenced cells had more mitochondrial mass than that in control cells. More importantly, this difference was significantly increased by hypoxia treatment (1% O_2_) for 24 h. Since mitochondrial mass is closely related with oxygen consumption in metabolically active cells, we further measured and compared the oxygen consumption of NAC1 silenced and control cells with or without hypoxia treatment (1% O_2_) for 24 h in Oxyograph chambers. **Figure 1B** shows that when allowed to respire in full media, HeLa and SKOV3 cells with silencing of NAC1 consumed more oxygen than control cells. After hypoxia treatment, this difference was further enhanced as the NAC1 silenced cells failed to effectively reduce mitochondrial oxygen consumption, reflecting the obtained results of mitochondrial mass evaluation (**Figure 1A**). When incubated in glutamine-free media, NAC1 silenced cells consumed more oxygen consumption than control cells in hypoxia in part due to Crabtree effect (**Figure 1B**). Deprived of glucose in the cells showed a strong increase in oxygen consumption in all groups, partly due to increased glutaminolysis, but further reflected the greater capacity of the NAC1 silenced cells for oxygen consumption (**Figure 1B**). Furthermore, the inner mitochondrial transmembrane potential (ΔΨm) in control cells and NAC1 siRNA cells were determined by JC-1 fluorescence. **Figure 1C** shows that HeLa and SKOV3 cells with silencing of NAC1 expression disrupted the mitochondrial integrity, as proved by a loss of mitochondrial membrane potential (ΔΨm) compared with control cells especially after hypoxia treatment. Taken together, these results indicate that silencing of NAC1 expression activated mitochondrial function and, importantly, failed to properly downregulate their mitochondrial respiration and to control mitochondrial membrane potential (ΔΨm) in hypoxia.

### NAC1-PDK3 Pathway Controls Mitochondrial Respiration in Hypoxia

**FIGURE 2:**
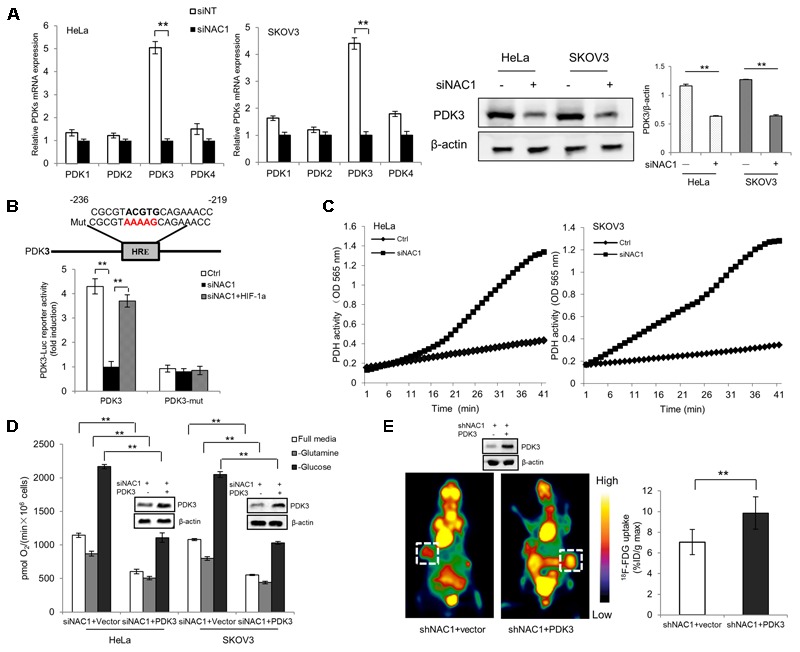
Nucleus accumbens-1 controls mitochondrial respiration via pyruvate dehydrogenase kinase 3 (PDK3) in hypoxia. **(A)** HeLa and SKOV3 cells transfected with a non-targeting RNA (Ctrl) or NAC1-targeted siRNA were incubated in hypoxia (1% O_2_) for 24 h. PDK1–4 isoforms mRNA expression levels were measured by using quantitative reverse transcriptase-polymerase chain reaction (RT-PCR), and plotted after normalization. Bars are mean ± SD of three independent experiments. HeLa and SKOV3 cells were subjected to same treatment as above and PDK3 protein expression levels were examined by Western Blot, with β-actin as a loading control. Data shown are the representative of three identical experiments. **(B)** HeLa cells with silencing of NAC1 expression with or without HIF-1α plasmid were transfected with PDK3 or HRE mutated PDK3 (PDK3-mut) reporter constructs, 12 h later, the cells were then incubated in hypoxia (1% O_2_) for additional 24 h. Luciferase activity of the PDK3 promoter was measured by using the Dual-Luciferase Reporter Assay system, and plotted after normalization with the activity of Renilla luciferase. Bars are mean ± SD of three independent experiments. **(C)** HeLa and SKOV3 cells transfected with a non-targeting RNA (Ctrl) or NAC1-targeted siRNA were incubated in hypoxia (1% O_2_) for 24 h. PDH activity was measured by using PDH Assay Kit. Microplate-recorded data are representative of three independent experiments. **(D)** HeLa and SKOV3 cells with silencing of NAC1 expression were transfected with or without PDK3 plasmid for 12 h, and then exposed to hypoxia for additional 24 h. Oxygen consumption was measured in full, glucose-free, or glutamine-free media by using an oxygen electrode, and normalized to cell numbers. Bars are mean ± SD of three independent experiments. **(E)** Mice inoculated with HeLa cells expressing NAC1-targeted shRNA were infected with or without PDK3 virus. Glucose uptake (^18^F-FDG) was analyzed by microPET. Images of white squares point to tumors. Data are shown as mean ± SD of *n* = 5 mice per group. ^∗∗^*p* < 0.01.

Pyruvate, the end product of glycolysis, may be converted into acetyl coenzyme A (acetyl-CoA) in the mitochondria by PDH for aerobic respiration, or to lactate by the lactate dehydrogenase (LDH) in cancer cells. In light of our previous report NAC1 can promote hypoxia-induced glycolysis via HIF-1α pathway (Zhang et al., 2017), and HIF-1α could modulate metabolic switches through upregulation of Pyruvate dehydrogenase kinases (PDKs) (Lu et al., 2008; Sun et al., 2009; Prigione et al., 2014; Dupuy et al., 2015; Mazar et al., 2016), we aimed to determine whether the expression PDKs (PDK1–4) in hypoxia was regulated by NAC1. Quantitative RT-PCR analysis showed that the NAC1 silenced cells correlated with the reduced expression of PDK3 mRNA in hypoxia, whereas other related isoforms exhibited little or modest changes (**Figure 2A**). Results were confirmed at the protein level (**Figure 2A**). To further investigate whether NAC1-induced PDK3 expression in hypoxia was mediated by HIF-1α at transcriptional level, mutation of the HRE sequence in PDK3 [binding elements of HIF-1α, (Lu et al., 2008)] completely abolished promoter activity in control cells under hypoxia, whereas re-expression of HIF-1α in NAC1 silenced cells could rescue PDK3 promoter activity (**Figure 2B**). We also performed chromatin IP to test whether NAC1-HDAC4-HIF-1α bound to the promoter of PDK3. We found that binding of endogenous NAC1, HDAC4, and HIF-1α were observed for PDK3 promoter and were further enriched upon hypoxic treatment (Supplementary Figure S1). The enrichment of NAC1, HDAC4 and HIF-1α binding were clearly decreased in the NAC1 silenced cells, and these differences were significantly decreased by hypoxia treatment (Supplementary Figure S1). In agreement with the PDK3 expression downregulation, the enzymatic activity of PDH was significantly enhanced in NAC1 silenced cells under hypoxia (**Figure 2C**). **Figure 2D** shows that in the hypoxic cells with silencing of NAC1 expression, re-expression of PDK3 caused a greater decrease in oxygen consumption. In addition, we inoculated mice subcutaneously with the NAC1 shRNA HeLa cells expressing either a control retrovirus construct or retrovirus PDK3 construct, and then examined glucose uptake. **Figure 2E** shows that ^18^F-FDG uptake was significantly increased in the tumors with infection retrovirus PDK3 construct as analyzed by microPET. Taken together, these results indicate that NAC1 potently upregulates PDK3 expression via HIF-1α at transcriptional level, and consequently decreases PDH activity, leading to repression of mitochondrial respiration and increase of glycolytic flux in hypoxia.

### Silencing of NAC1 Expression Enhances Mitochondrial ROS Production in Hypoxia

**FIGURE 3:**
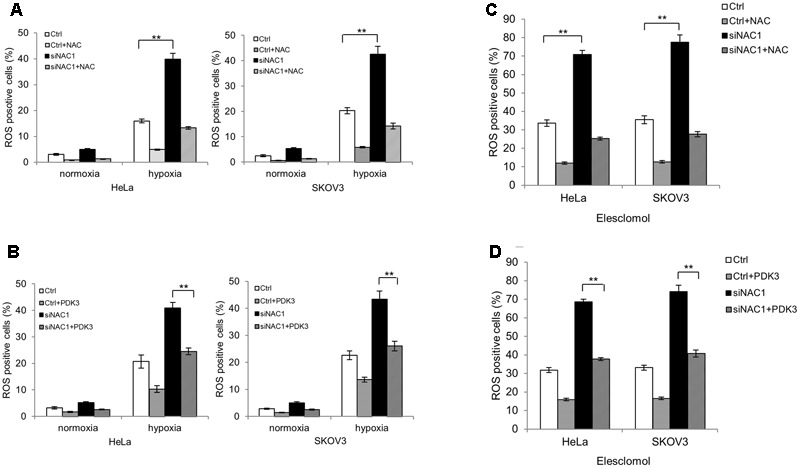
Silencing of NAC1 expression promotes reactive oxygen species (ROS) generation in hypoxic cancer cells. **(A)** HeLa and SKOV3 cells transfected with a non-targeting RNA (Ctrl) or NAC1-targeted siRNA were incubated for 24 h in the presence or absence of NAC (2 mM) under normoxia or hypoxia (1% O_2_) conditions, and the level of ROS was measured. Bars are mean ± SD of three independent experiments. **(B)** HeLa and SKOV3 cells with or without silencing of NAC1 expression were transfected with or without PDK3 plasmid for 12 h, and incubated in normoxia or hypoxia (1% O_2_) for additional 24 h, and the level of ROS was measured. Bars are mean ± SD of three independent experiments. **(C)** HeLa and SKOV3 cells transfected with a non-targeting RNA (Ctrl) or NAC1-targeted siRNA were treated with 300 nmol/L elesclomol for 12 h, and ROS production was assessed in the presence or absence of 2 mM NAC. Bars are mean ± SD of three independent experiments. **(D)** HeLa and SKOV3 cells with or without silencing of NAC1 expression were transfected with PDK3 plasmid for 24 h, and then treated with 300 nmol/L elesclomol for additional 12 h, the level of ROS was measured. Bars are mean ± SD of three independent experiments. ^∗∗^*p* < 0.01.

Next, we decided to explore whether the enhanced mitochondrial respiration in NAC1 silenced cells would lead to increased mitochondrial ROS generation in hypoxia. **Figures 3A,B** show that HeLa and SKOV3 cells with silencing of NAC1 expression increased ROS levels compared with control cells in hypoxia. Pretreatment of NAC1 silenced cells with antioxidant N acetylcysteine (NAC) or re-expression of PDK3 in cells with silencing of NAC1 expression greatly reduced the production of ROS in hypoxia (**Figures 3A,B**). Elesclomol, a strong inducer of oxidative stress, is always associated with hypoxic microenvironment (Barbi de Moura et al., 2012; Blackman et al., 2012). Then, we aimed to investigate whether mitochondrial ROS generation could be promoted by combination of NAC1 siRNA and elesclomol. Consistently, exposure of cells lacking NAC1 to elesclomol generated more ROS than control cells. Pretreatment of NAC1 silenced cells with antioxidant NAC or re-expression of PDK3 in cells with silencing of NAC1 expression also greatly reduced elesclomol-induced ROS production (**Figures 3C,D**). Our results suggest that the NAC1-PDK3 pathway protects hypoxic cancer cells from mitochondrial ROS production.

### Elevated ROS Levels in NAC1 Silenced Cells Contribute Them to Hypoxia-Induced Apoptosis

**FIGURE 4:**
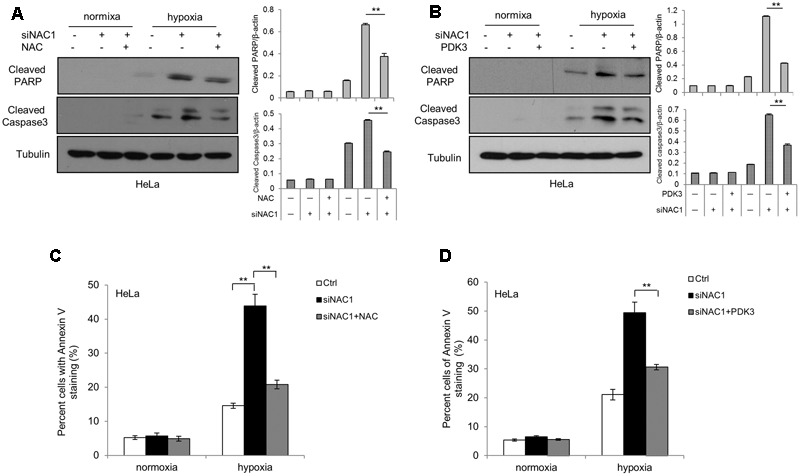
Suppression of ROS production shows decreased sensitivity to hypoxia-induced apoptosis. HeLa cells transfected with a non-targeting RNA (Ctrl) or NAC1-targeted siRNA were incubated for 24 h in the presence or absence of NAC (2 mM) under normoxia or hypoxia (1% O_2_) conditions; HeLa cells with silencing of NAC1 expression were transfected with or without PDK3 plasmid for 12 h, and incubated under normoxia or hypoxia (1% O_2_) conditions for additional 24 h. Apoptosis was determined by: **(A,B)** western blot analysis of cleaved PARP and cleaved caspase-3, with β-actin as a loading control and **(C,D)** flow cytometric analysis of Annexin V staining. Bars are mean ± SD of three independent experiments. ^∗∗^*p* < 0.01.

To explore the connection between ROS generation and cells’ adaptation to hypoxia, we first pretreated the NAC1 silenced HeLa cells with antioxidant NAC. NAC pretreatment attenuated the hypoxia-induced cell apoptosis in hypoxia, as indicated by decreases in the levels of cleaved Caspase-3 and cleaved PARP and in Annexin V staining (**Figures 4A,C**). To determine whether PDK3 down-regulation mediated ROS production in cancer cells with absence of NAC1 contribute to hypoxia-induced apoptosis, we transfected a Flag-PDK3 plasmid into HeLa cells with silencing of NAC1 expression. Ectopic expression of PDK3 regained resistance to hypoxia, as evidenced by blunted the activation of apoptosis induced by hypoxia, as indicated decreases in the levels of cleaved Caspase-3 and cleaved PARP and in Annexin V staining (**Figures 4B,D**). Taken together, these results indicate that ROS may serve as a necessary mediator of hypoxic adaption in NAC1 silenced cells, and decrease of ROS generation indeed partly protects cancer cells with absence of NAC1 from hypoxia-induced apoptosis.

### Silencing of NAC1 Expression Promotes the Antitumor Efficacy of Elesclomol

**FIGURE 5:**
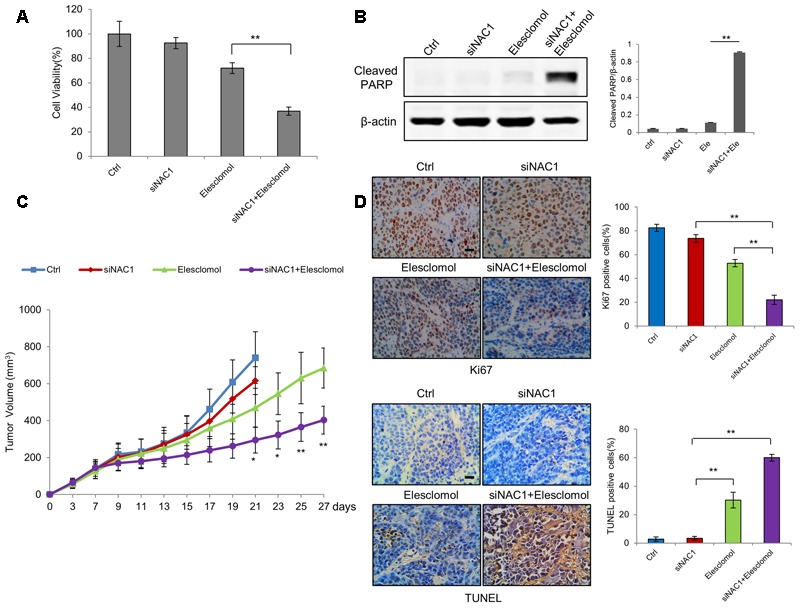
Silencing of NAC1 expression promotes anti-tumor activity of elesclomol *in vitro* and *in vivo.* **(A)** HeLa cells transfected with a non-targeting RNA (Ctrl) or NAC1-targeted siRNA were treated with 300 nmol/L elesclomol for 12 h. Cell viability was measured by MTT assay. Bars are mean ± SD of three independent experiments. **(B)** Apoptosis was determined by western blot analysis of cleaved PARP, with β-actin as a loading control. Data shown are the representative of three identical experiments. **(C)** The nude mice inoculated with HeLa cells were randomly divided into four groups: (1) control group; (2) siNAC1 group; (3) elesclomol group; (4) elesclomol/siNAC1 group. The tumor sizes were measured with calipers every other day up to 4 weeks. ^∗^*P <* 0.05, ^∗∗^*P <* 0.01, elesclomol/siNAC1 vs elesclomol. Data are shown as mean ± SD of *n* = 5 mice per group. **(D)** Images represent immuno-histochemical staining of Ki67 and TUNEL in tumor specimens from different groups. Bars are mean ± SD (*n* = 5). The scale bar represents 25 μm.

Finally, we tested whether enhancement of mitochondrial ROS generation by NAC1 siRNA and elesclomol exerted potent antitumor activities. The combination of NAC1 siRNA and elesclomol-induced cell viability inhibition and apoptosis more effectively than each treatment did alone (**Figures 5A,B**). To further explore the effect of silencing of NAC1 expression on antitumor efficacy of elesclomol, we aimed to determine whether NAC1 siRNA treatment could sensitize HeLa xenograft model to elesclomol. We observed that NAC1 siRNA or elesclomol alone showed moderate reduction in growth of HeLa xenografts (**Figure 5C**). In comparison, the combination of NAC1 siRNA and elesclomol showed more effectively in inhibiting tumor growth than single agent alone, as presented by the marked differences in the tumor volume (**Figure 5C**). Concomitantly, combined treatment of NAC1 siRNA with elesclomol caused a significantly more increase in apoptosis than single treatment did alone, as evidenced by the increased numbers of TUNEL positive cells (**Figure 5D**). By contrast, combined treatment of NAC1 siRNA with elesclomol caused a significantly more decrease in proliferation than single treatment did alone, as evidenced by the decreased numbers of Ki67 positive cells (**Figure 5D**). Altogether, these results suggest that silencing of NAC1 expression can promote the antitumor activity of elesclomol *in vitro* and *in vivo*.

## Discussion

The switch of hypoxia-induced cellular metabolism from mitochondrial respiration to glycolysis not only contributes to adaptation of cancer cells under hypoxic conditions but also is required for them to survive and propagate. Although NAC1 has been considered as an activator of glycolysis, however, how NAC1 mediates mitochondrial oxidative respiration in hypoxia is not clear. In this study, we demonstrated that silencing of NAC1 expression increased mitochondrial mass, oxygen consumption, and excessive ROS production which is associated with an increased vulnerability to apoptotic cell death in hypoxia (**Figures 1, 3A,B, 4**).

Pyruvate dehydrogenase complex, an important gatekeeper enzyme, is responsible for catalyzing the oxidative decarboxylation of pyruvate to acetyl-CoA, and thereby supplies the procession of TCA cycle. PDKs negatively regulate PDH activity through phosphorylation and drive pyruvate into more lactate production (Patel and Korotchkina, 2001; Roche and Hiromasa, 2007). By checking the entire four isoenzymes (PDK1–4), we demonstrated that NAC1 can induce PDK3 but not PDK-1, -2 or -4 expression and ensure efficient blockage of PDH activity under hypoxia (**Figures 2A,C**). Overexpression of PDK3 could reduce oxygen consumption and excessive ROS production in hypoxic NAC1 absent cancer cells, whereas increase glucose uptake *in vivo* (**Figures 2D,E, 3B**). One study showed that cancer cells with mitochondrial respiration defects exhibit a survival advantage and withstand metabolic stress (Pelicano et al., 2006). Indeed, in this study we demonstrated that silencing of NAC1 expression promoted mitochondrial respiration and increased vulnerability to cell death in hypoxia, and survival advantage was reversed when PDK3 was over-expressed in NAC1 absent cancer cells (**Figures 4B,D**). Interestingly, it is well known that high concentration of pyruvate can inhibit the PDK-1, -2, and -4 activity, but not PDK3 (Bowker-Kinley et al., 1998; Baker et al., 2000). The results of this study were consistent with these findings, as NAC1-overexpressed cancer cells would result in the accumulation of pyruvate in hypoxia (Zhang et al., 2017), and NAC1 can induce PDK3 but not PDK-1, -2, or -4 expression in hypoxia (**Figure 2A**). Therefore, NAC1/PDK3 axis can guarantee cancer cells continuously shutdown of mitochondrial respiration when face hypoxic stress. Binding elements (HRE) in the promoter regions PDK3 gene has a key role in PDK3 transcriptional activity in the context of HIF-1α (Lu et al., 2008). Indeed, the use of HRE mutation in PDK3 supports the role of HIF-1α in the NAC1-promoted transcriptional activity of PDK3 in hypoxia (**Figure 2B**).

Elesclomol, a first-in-class mitochondrial-targeted agent that interferes with the electron flow in cancer cell to promote mitochondrial ROS production has entered the clinical trials (O’Day et al., 2009). Nevertheless, the induction of hypoxic microenvironment by elesclomol diminishes its efficacy (Bair et al., 2010; Barbi de Moura et al., 2012). Our study showed that combined silencing of NAC1 expression along with elesclomol could result in huge production of ROS, reverse therapeutic resistance and enhance the efficacy of pro-oxidative agent elesclomol (**Figure 5**), suggesting that NAC1 appears to be a potential target to improve pro-oxidative therapy in human cancers such as elesclomol.

Previous studies including our own have demonstrated that NAC1 is an important oncogene with multiple functions. One study indicated that NAC1 can form 300–500 kDa complexes in tumor cells (Nakayama et al., 2016), however, limited interaction partners of NAC1 like HDAC4, Miz1 have been reported (Stead and Wright, 2014; Zhang et al., 2017) and it is unclear how they work together. Efforts to further identify other interaction partners of the NAC1 in different types of tumor cells by both IP-Mass spec and experimental examination will provide a full understanding of NAC1 repression.

In summary, this study indicates that the inability of cancer cells to downregulate mitochondrial respiration and excessive ROS production upon NAC1 silenced correlates with increased cell apoptosis in hypoxia, and demonstrates that this previously unrecognized function of NAC1 involved in depressing mitochondrial respiration in hypoxia is mediated through the HIF-1α-PDK3 axis. These findings provide a novel insight for understanding oncogenic role of NAC1, and suggest that targeting NAC1 may be employed as a pivotal strategy for cancer progression and treatment.

## Conclusion

This is the first report to show the critical role of NAC1 in suppressing mitochondrial function in hypoxia and inhibiting hypoxia-induced apoptosis. We show that suppression of mitochondrial function in hypoxia by NAC1 is mediated though inducing expression of PDK3 by HIF-1α at the transcriptional level, thereby inactivating PDH and blocking the mitochondrial respiration. Furthermore, we provide the *in vivo* data supporting the roles of NAC1-PDK3 pathway in promoting glycolysis, and in modulating therapeutic response. These findings not only provide the NAC1-PDK3 axis as a novel molecular pathway involved in regulating mitochondrial function and survival of hypoxic tumor cells, but also suggest the potential of targeting NAC1 for enhancing ROS production as an effective strategy to improve pro-oxidative therapy in human cancer such as elesclomol.

## Author Contributions

YZ and YC: Contributed in conception and design. Y-JR, X-HW, CJ, Y-DG, X-JL, X-RL, Q-HX, J-MY, YC, and YZ: Contributed in development of methodology. Y-JR, X-HW, CJ, Y-DG, X-JL, X-RL, Q-HX, L-CG, H-HZ, and W-DZ: Contributed in acquisition of data (provided animals, provided facilities, etc.). Y-JR, Y-DG, Z-JM, J-MY, YC, and YZ: Contributed in analysis and interpretation of data. J-MY, YC, and YZ: Contributed in writing, review, and/or revision of the manuscript. J-MY, YC, and YZ: Contributed in administrative, technical, or material support. J-MY, YC, and YZ: Contributed in study supervision. L-CG and W-DZ: Other (reviewed the pathology slides).

## Conflict of Interest Statement

The authors declare that the research was conducted in the absence of any commercial or financial relationships that could be construed as a potential conflict of interest.

## References

[B1] Annibaldi A. Widmann C. (2010). Glucose metabolism in cancer cells. *Curr. Opin. Clin. Nutr. Metab. Care* 13 466–470. 10.1097/MCO.0b013e32833a5577 20473153

[B2] Bair J. S. Palchaudhuri R. Hergenrother P. J. (2010). Chemistry and biology of deoxynyboquinone, a potent inducer of cancer cell death. *J. Am. Chem. Soc.* 132 5469–5478. 10.1021/ja100610m 20345134

[B3] Baker J. C. Yan X. Peng T. Kasten S. Roche T. E. (2000). Marked differences between two isoforms of human pyruvate dehydrogenase kinase. *J. Biol. Chem.* 275 15773–15781. 10.1074/jbc.M909488199 10748134

[B4] Barbi de Moura M. Vincent G. Fayewicz S. L. Bateman N. W. Hood B. L. Sun M. (2012). Mitochondrial respiration–an important therapeutic target in melanoma. *PLOS ONE* 7:e40690. 10.1371/journal.pone.0040690 22912665 PMC3422349

[B5] Blackman R. K. Cheung-Ong K. Gebbia M. Proia D. A. He S. Kepros J. (2012). Mitochondrial electron transport is the cellular target of the oncology drug elesclomol. *PLOS ONE* 7:e29798. 10.1371/journal.pone.0029798 22253786 PMC3256171

[B6] Bowker-Kinley M. M. Davis W. I. Wu P. Harris R. A. Popov K. M. (1998). Evidence for existence of tissue-specific regulation of the mammalian pyruvate dehydrogenase complex. *Biochem. J.* 329(Pt 1) 191–196. 10.1042/bj3290191 9405293 PMC1219031

[B7] Dupuy F. Tabaries S. Andrzejewski S. Dong Z. Blagih J. Annis M. G. (2015). PDK1-dependent metabolic reprogramming dictates metastatic potential in breast cancer. *Cell Metab.* 22 577–589. 10.1016/j.cmet.2015.08.007 26365179

[B8] Fukuda R. Zhang H. Kim J. W. Shimoda L. Dang C. V. Semenza G. L. (2007). HIF-1 regulates cytochrome oxidase subunits to optimize efficiency of respiration in hypoxic cells. *Cell* 129 111–122. 10.1016/j.cell.2007.01.047 17418790

[B9] Jinawath N. Vasoontara C. Yap K. L. Thiaville M. M. Nakayama K. Wang T. L. (2009). NAC-1, a potential stem cell pluripotency factor, contributes to paclitaxel resistance in ovarian cancer through inactivating Gadd45 pathway. *Oncogene* 28 1941–1948. 10.1038/onc.2009.37 19305429 PMC2679096

[B10] Kluza J. Jendoubi M. Ballot C. Dammak A. Jonneaux A. Idziorek T. (2011). Exploiting mitochondrial dysfunction for effective elimination of imatinib-resistant leukemic cells. *PLOS ONE* 6:e21924. 10.1371/journal.pone.0021924 21789194 PMC3138741

[B11] Kroemer G. Pouyssegur J. (2008). Tumor cell metabolism: cancer’s Achilles’ heel. *Cancer Cell* 13 472–482. 10.1016/j.ccr.2008.05.005 18538731

[B12] Lu C. W. Lin S. C. Chen K. F. Lai Y. Y. Tsai S. J. (2008). Induction of pyruvate dehydrogenase kinase-3 by hypoxia-inducible factor-1 promotes metabolic switch and drug resistance. *J. Biol. Chem.* 283 28106–28114. 10.1074/jbc.M803508200 18718909 PMC2661383

[B13] Mazar J. Qi F. Lee B. Marchica J. Govindarajan S. Shelley J. (2016). MicroRNA 211 functions as a metabolic switch in human melanoma cells. *Mol. Cell. Biol.* 36 1090–1108. 10.1128/MCB.00762-15 26787841 PMC4800793

[B14] Nakayama K. Nakayama N. Davidson B. Sheu J. J. Jinawath N. Santillan A. (2006). A BTB/POZ protein, NAC-1, is related to tumor recurrence and is essential for tumor growth and survival. *Proc. Natl. Acad. Sci. U.S.A.* 103 18739–18744. 10.1073/pnas.0604083103 17130457 PMC1693732

[B15] Nakayama K. Nakayama N. Wang T. L. Shih I. E. M. (2007). NAC-1 controls cell growth and survival by repressing transcription of Gadd45GIP1, a candidate tumor suppressor. *Cancer Res.* 67 8058–8064. 10.1158/0008-5472.CAN-07-1357 17804717

[B16] Nakayama K. Rahman M. T. Rahman M. Yeasmin S. Ishikawa M. Katagiri A. (2010). Biological role and prognostic significance of NAC1 in ovarian cancer. *Gynecol. Oncol.* 119 469–478. 10.1016/j.ygyno.2010.08.031 20869761

[B17] Nakayama N. Kato H. Sakashita G. Nariai Y. Nakayama K. Kyo S. (2016). Protein complex formation and intranuclear dynamics of NAC1 in cancer cells. *Arch. Biochem. Biophys.* 606 10–15. 10.1016/j.abb.2016.07.007 27424155

[B18] O’Day S. Gonzalez R. Lawson D. Weber R. Hutchins L. Anderson C. (2009). Phase II, randomized, controlled, double-blinded trial of weekly elesclomol plus paclitaxel versus paclitaxel alone for stage IV metastatic melanoma. *J. Clin. Oncol.* 27 5452–5458. 10.1200/JCO.2008.17.1579 19826135

[B19] Papandreou I. Cairns R. A. Fontana L. Lim A. L. Denko N. C. (2006). HIF-1 mediates adaptation to hypoxia by actively downregulating mitochondrial oxygen consumption. *Cell Metab.* 3 187–197. 10.1016/j.cmet.2006.01.012 16517406

[B20] Patel M. S. Korotchkina L. G. (2001). Regulation of mammalian pyruvate dehydrogenase complex by phosphorylation: complexity of multiple phosphorylation sites and kinases. *Exp. Mol. Med.* 33 191–197. 10.1038/emm.2001.32 11795479

[B21] Pelicano H. Xu R. H. Du M. Feng L. Sasaki R. Carew J. S. (2006). Mitochondrial respiration defects in cancer cells cause activation of Akt survival pathway through a redox-mediated mechanism. *J. Cell Biol.* 175 913–923. 10.1083/jcb.200512100 17158952 PMC2064701

[B22] Perez-Torrado R. Yamada D. Defossez P. A. (2006). Born to bind: the BTB protein-protein interaction domain. *Bioessays* 28 1194–1202. 10.1002/bies.20500 17120193

[B23] Prigione A. Rohwer N. Hoffmann S. Mlody B. Drews K. Bukowiecki R. (2014). HIF1alpha modulates cell fate reprogramming through early glycolytic shift and upregulation of PDK1-3 and PKM2. *Stem Cells* 32 364–376. 10.1002/stem.1552 24123565 PMC5730046

[B24] Roche T. E. Hiromasa Y. (2007). Pyruvate dehydrogenase kinase regulatory mechanisms and inhibition in treating diabetes, heart ischemia, and cancer. *Cell. Mol. Life Sci.* 64 830–849. 10.1007/s00018-007-6380-z 17310282 PMC11136253

[B25] Shih I. E. M. Nakayama K. Wu G. Nakayama N. Zhang J. Wang T. L. (2011). Amplification of the ch19p13.2 *NACC*1 locus in ovarian high-grade serous carcinoma. *Mod. Pathol.* 24 638–645. 10.1038/modpathol.2010.230 21240255 PMC3085564

[B26] Stead M. A. Wright S. C. (2014). Nac1 interacts with the POZ-domain transcription factor, Miz1. *Biosci. Rep.* 34:e00110. 10.1042/BSR20140049 24702277 PMC4046208

[B27] Sun W. Zhou S. Chang S. S. McFate T. Verma A. Califano J. A. (2009). Mitochondrial mutations contribute to HIF1alpha accumulation via increased reactive oxygen species and up-regulated pyruvate dehydrogenase kinase 2 in head and neck squamous cell carcinoma. *Clin. Cancer Res.* 15 476–484. 10.1158/1078-0432.CCR-08-0930 19147752 PMC2729126

[B28] Ueda S. M. Yap K. L. Davidson B. Tian Y. Murthy V. Wang T. L. (2010). Expression of fatty acid synthase depends on NAC1 and is associated with recurrent ovarian serous carcinomas. *J. Oncol.* 2010:285191. 10.1155/2010/285191 20508725 PMC2873657

[B29] Vander Heiden M. G. Cantley L. C. Thompson C. B. (2009). Understanding the Warburg effect: the metabolic requirements of cell proliferation. *Science* 324 1029–1033. 10.1126/science.1160809 19460998 PMC2849637

[B30] Wang J. Rao S. Chu J. Shen X. Levasseur D. N. Theunissen T. W. (2006). A protein interaction network for pluripotency of embryonic stem cells. *Nature* 444 364–368. 10.1038/nature05284 17093407

[B31] Yap K. L. Fraley S. I. Thiaville M. M. Jinawath N. Nakayama K. Wang J. (2012). NAC1 is an actin-binding protein that is essential for effective cytokinesis in cancer cells. *Cancer Res.* 72 4085–4096. 10.1158/0008-5472.CAN-12-0302 22761335 PMC3421062

[B32] Yeasmin S. Nakayama K. Rahman M. T. Rahman M. Ishikawa M. Katagiri A. (2012). Biological and clinical significance of NAC1 expression in cervical carcinomas: a comparative study between squamous cell carcinomas and adenocarcinomas/adenosquamous carcinomas. *Hum. Pathol.* 43 506–519. 10.1016/j.humpath.2011.05.021 21889186

[B33] Zhang Y. Cheng Y. Ren X. Hori T. Huber-Keener K. J. Zhang L. (2012a). Dysfunction of nucleus accumbens-1 activates cellular senescence and inhibits tumor cell proliferation and oncogenesis. *Cancer Res.* 72 4262–4275. 10.1158/0008-5472.CAN-12-0139 22665267 PMC3614094

[B34] Zhang Y. Cheng Y. Ren X. Zhang L. Yap K. L. Wu H. (2012b). NAC1 modulates sensitivity of ovarian cancer cells to cisplatin by altering the HMGB1-mediated autophagic response. *Oncogene* 31 1055–1064. 10.1038/onc.2011.290 21743489 PMC3275651

[B35] Zhang Y. Ren Y. J. Guo L. C. Ji C. Hu J. Zhang H. H. (2017). Nucleus accumbens-associated protein-1 promotes glycolysis and survival of hypoxic tumor cells via the HDAC4-HIF-1alpha axis. *Oncogene* 36 4171–4181. 10.1038/onc.2017.51 28319066 PMC5537617

