# Supplementary material for: Silencing of NAC1 Expression Induces Cancer Cells Oxidative Stress in Hypoxia and Potentiates the Therapeutic Activity of Elesclomol
Source: Front Pharmacol. 2017 Nov 7;8:804. doi: 10.3389/fphar.2017.00804 (PMC5681923; doi:10.3389/fphar.2017.00804)
Supplement: Supplementary file 1 [file Data_Sheet_1.DOCX]

**Legends to Supplement Figure**

**Figure S1. NAC1-HDAC4-HIF-1α bound to the promoter of PDK3 in hypoxia.** HeLa cells transfected with a non-targeting RNA (Ctrl) or NAC1-targeted siRNA were incubated in normoxia or hypoxia (1% O_2_) for 18h before crosslinking. Chromatin IP was performed using anti-NAC1 **(A)**, anti-HDAC4 **(B)**, anti-HIF-1α **(C),** and IgG (negative control) antibodies. Primers of PDK3 HRE promoter were designed. Results were generated by qPCR and are presented as percentage bound/input (%). Bars are mean ±s.d. of three independent experiments.
