# Supplementary figures and images for: Silencing of NAC1 Expression Induces Cancer Cells Oxidative Stress in Hypoxia and Potentiates the Therapeutic Activity of Elesclomol
Source: Front Pharmacol. 2017 Nov 7;8:804. doi: 10.3389/fphar.2017.00804 (PMC5681923; doi:10.3389/fphar.2017.00804)

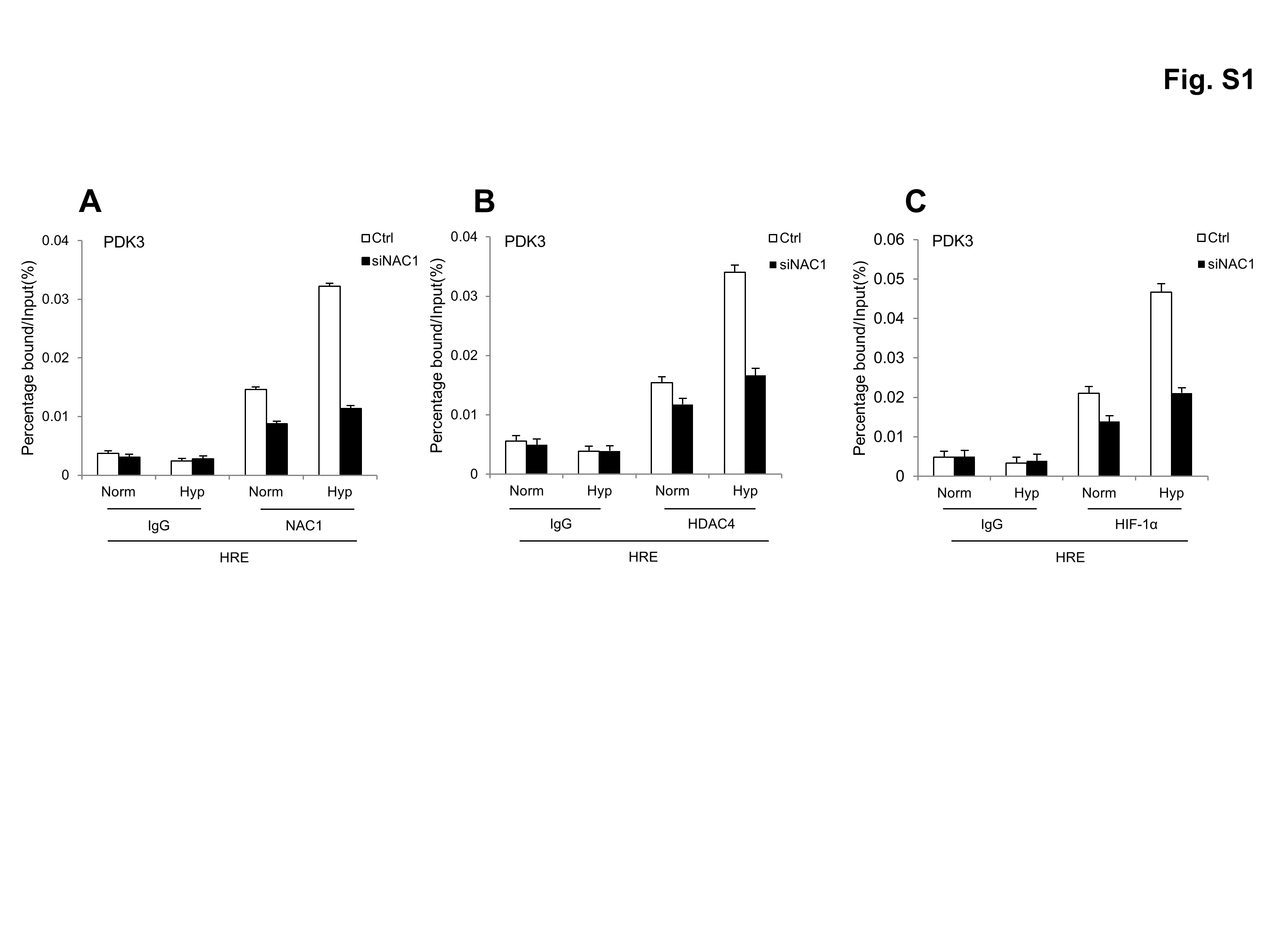

Supplement: Supplementary file 2 [file Image_1.TIF]
